# Complete Genome Sequences of Evolved Arsenate-Resistant *Metallosphaera sedula* Strains

**DOI:** 10.1128/genomeA.01142-15

**Published:** 2015-10-01

**Authors:** Chenbing Ai, Samuel McCarthy, Wendy Schackwitz, Joel Martin, Anna Lipzen, Paul Blum

**Affiliations:** a School of Biological Sciences, University of Nebraska-Lincoln, Lincoln, Nebraska, USA; b School Of Minerals Processing and Bioengineering, Central South University, Hunan, China; c U.S. Department of Energy-Joint Genome Institute, Walnut Creek, California, USA

## Abstract

*Metallosphaera sedula* is a thermoacidophilic crenarchaeote with a 2.19-Mb genome. Here, we report the genome sequences of several evolved derivatives of *M. sedula* generated through adaptive laboratory evolution for enhanced arsenate resistance.

## GENOME ANNOUNCEMENT

*Metallosphaera sedula* is a thermoacidophilic crenarchaeote that grows optimally at 75°C and pH 2.0 and is capable of lithoautotrophy (1, 2). Its ability to oxidize iron and sulfur (2) is used in biomining, and this organism has been used to extract copper from low-grade copper sulfide ores and tailings, especially by heap bioleaching (3). The oxidation of copper sulfide ores is an exothermic process that can elevate temperatures inside mineral heaps to 60 to ~80°C (4). These temperatures are lethal to mesophilic acidophiles and moderate the thermoacidophiles used in biomining. However, as an extreme thermoacidophile, *M. sedula* is able to withstand and continue bioleaching at these high temperatures.

Arsenic is very prevalent in low-grade copper sulfide ores and tailings, and during biomining, it is released from the ores along with the other metals. Extreme thermoacidophiles used in bioleaching, such as *M. sedula*, are very sensitive to arsenic (1, 5). Bioinformatics analysis has shown that previously studied arsenic resistance pathways in biomining mesophiles and moderate thermoacidophiles, such as the *ars* operon (6, 7), are not present in these organisms. Therefore, arsenate-resistant *M. sedula* strains would be beneficial for the bioprocessing of arsenic-bearing copper sulfide ores and tailings. Four arsenate-adapted derivatives of *M. sedula* (DSM 5348) and the copper-resistant *M. sedula* strain CuR1 (8) were isolated through adaptive laboratory evolution, involving extensive passage during selection for the biological trait of increased arsenate resistance (unpublished data). Here, we report the complete genome sequence of arsenate-resistant isolates derived from *M. sedula* DSM 5348, named ARS120-1 and ARS120-2, and those derived from *M. sedula* strain CuR1 (8), named ARS50-1 and ARS50-2.

High-molecular-weight genomic DNA was prepared from clonal cultures of the *M. sedula* strains, as described previously (2, 9). The integrity and purity of the DNA samples were verified by spectroscopic measurements at 260/280 and 260/230 nm and confirmed by agarose gel electrophoresis. DNA and RNA library preparation was conducted using the Joint Genome Institute (JGI)’s automated process with a Biomek FX robot. The samples were sheared using a Covaris E210 sonicator, followed by end repair and phosphorylation. Fragments ranging from 100 to 500 bp were selected for sequencing using an automated solid-phase reversible immobilization selection system. The addition of 3′ terminal adenine was made to the fragments, followed by adaptor sequence ligation. Genome sequencing of the libraries was done using an Illumina HiSeq 2500, generating paired-end 100-bp reads. Samples were applied to a 25-Gb 2 × 100 channel that gave 1 Gb of sequence information per sample (500× coverage). The sequences were mapped to the *M. sedula* DSM 5348 reference genome (GenBank accession no. CP000682.1) using Bowtie2 (version 2.1.0) and SAMtools (version 1.0).

### Nucleotide sequence accession numbers.

The genome sequences of these evolved arsenate-resistant isolates of *M. sedula* DSM 5348, named ARS120-1 and ARS120-2, and *M. sedula* strain CuR1 (8), named ARS50-1 and ARS50-2, have been deposited in GenBank under accession numbers CP012174, CP012175, CP012172, and CP012173, respectively.
